# Scalable and automated CRISPR-based strain engineering using droplet microfluidics

**DOI:** 10.1038/s41378-022-00357-3

**Published:** 2022-03-15

**Authors:** Kosuke Iwai, Maren Wehrs, Megan Garber, Jess Sustarich, Lauren Washburn, Zachary Costello, Peter W. Kim, David Ando, William R. Gaillard, Nathan J. Hillson, Paul D. Adams, Aindrila Mukhopadhyay, Hector Garcia Martin, Anup K. Singh

**Affiliations:** 1 Technology Division, DOE Joint BioEnergy Institute, Emeryville, CA 94608 USA; 2 Biotechnology and Bioengineering Department, Sandia National Laboratories, Livermore, CA 94550 USA; 3 Biofuels and Bioproducts Division, DOE Joint BioEnergy Institute, Emeryville, CA 94608 USA; 4 Biological Systems and Engineering Division, Lawrence Berkeley National Laboratory, Berkeley, CA 94720 USA; 5 Molecular Biophysics and Integrated Bioimaging Division, Lawrence Berkeley National Laboratory, Berkeley, CA 94720 USA; 6 Department of Bioengineering, University of California, Berkeley, CA 94720 USA; 7 BCAM, Basque Center for Applied Mathematics, Bilbao, 48009 Spain

**Keywords:** Engineering, Microfluidics

## Abstract

We present a droplet-based microfluidic system that enables CRISPR-based gene editing and high-throughput screening on a chip. The microfluidic device contains a 10 × 10 element array, and each element contains sets of electrodes for two electric field-actuated operations: electrowetting for merging droplets to mix reagents and electroporation for transformation. This device can perform up to 100 genetic modification reactions in parallel, providing a scalable platform for generating the large number of engineered strains required for the combinatorial optimization of genetic pathways and predictable bioengineering. We demonstrate the system’s capabilities through the CRISPR-based engineering of two test cases: (1) disruption of the function of the enzyme galactokinase (*galK*) in *E. coli* and (2) targeted engineering of the glutamine synthetase gene (*glnA*) and the blue-pigment synthetase gene (*bpsA*) to improve indigoidine production in *E. coli*.

## Introduction

The CRISPR/Cas9 (clustered regularly interspaced short palindromic repeats and its associated protein, Cas9) system has proven to be a powerful tool for genome engineering in organisms, both eukaryotes and prokaryotes. Recombineering is a method of genetic engineering relying on short 50-base pair homologous recombination. It allows the precise insertion, deletion, or alteration of any sequence and is not dependent on the location of restriction sites that typically limit genetic engineering in bacterial systems such as *E. coli*. Multiplex automated genome engineering (MAGE) was developed to simultaneously introduce many chromosomal changes in a combinatorial fashion across a population of cells^1^. In *E. coli*, the CRISPR/Cas9 system has been recently coupled to λ Red oligo recombineering to improve its efficiency^2^. CRISPR-MAGE exploits intrinsic negative selection against the wild type of CRISPR/Cas9 to improve the performance of MAGE for small genome modifications such as codon substitution or translation control elements^3^. The system is based on two curable plasmids that encode optimized versions of both systems: λ Red recombineering and CRISPR/Cas9. However, realization of the high-throughput engineering potential of MAGE and CRISPR-MAGE requires automated instrumentation, and the robotic workstations that integrate the requisite unit operations are expensive and not accessible to most researchers.

Using micro-scale droplets as reaction chambers has proven to be a powerful approach to improve the throughput of synthetic biology experiments^4^, which require numerous design-build-test-learn cycles to achieve the target strain engineering goal. Such iterations are challenging in conventional benchtop procedures due to the limited throughput and manual operation. The benefits of this approach include faster reactions because of the small dimensions, low reagent consumption (enabling more reactions), and better control of the experimental process^5^. A number of microfluidic systems have been developed, including flow-based droplet microfluidics that rely on pressure-driven flow and digital microfluidics (DMF) that use an electrowetting-on-dielectric (EWOD) mechanism^4,6–10^.

**Table 1:** Comparison of our method to a traditional method

	Cuvette-based electroporation	Our approach
Throughput	Limited to 1–96 samples	100 or more
Minimum sample volume	20 µl or more	2 µl or less
Individual well addressability	No	Yes
Cell type	Multiple	Multiple

**Fig. 1:**
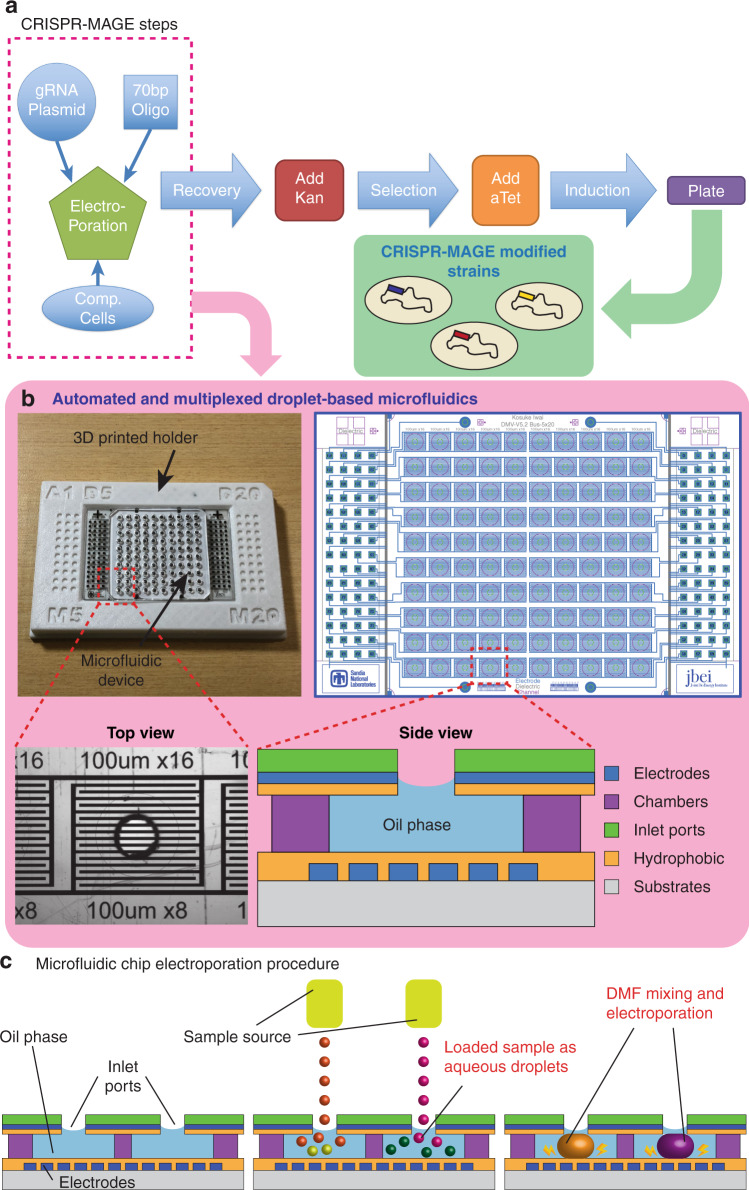
The microfluidic chip enables CRISPR-MAGE recombineering in an automated and multiplexed manner. **a** CRISPR-MAGE steps. The ones inside the box are performed on the chip. Cells are removed from the chip after the recovery step for induction and plating. **b** The microfluidic chip in a 3D printed holder (left), the electrode pattern (right), a top-view of an individual well, and a side-view schematic of a well. The chip is designed to contain 100 discrete reaction chambers with individually addressable electrodes for multiplexed CRISPR-MAGE recombineering, and its 384-well format design can be interfaced with lab automation equipment. **c** Droplets containing plasmids and cells are dispensed into each chamber through the inlet port, mixed by electrowetting, and electroporated by applying a voltage pulse

Here, we present a microfluidic platform for the miniaturization and automation of CRISPR-MAGE. The device has 100 wells, the bottom of each containing a set of electrodes for carrying out two functions: the electrowetting-based merger of droplets and electroporation for the transformation of cells (Fig. 1). The chip configuration uses a 384-well template and is easily integratable with liquid-handling robots (e.g., Labcyte Echo, iDOT, Hamilton, Tecan, OpenTrons, etc.), for automated sample input. The microfluidic chip is made with commonly used processes and materials, facilitating adoption by nonexperts in microfluidics. The microfluidic chip was used to perform targeted genomic changes in *E. coli* through CRISPR-based MAGE (CRMAGE^3^) recombineering in an automated fashion for two test cases. The first was to disrupt the function of the enzyme galactokinase (*galK*) to demonstrate CRMAGE. We then targeted engineering of the glutamine synthetase gene (*glnA*) and blue-pigment synthetase (*bpsA*) enzyme to improve indigoidine production. Indigoidine is a nonribosomal peptide with potential applications as a dye, antioxidant and antimicrobial compound. It is a viable alternative to indigo, currently one of the most common blue dyes used in the fabric industry. Indigo is derived from its precursor indican (present in plant leaves), but its production involves harsh reagents and environmentally detrimental waste streams. Indigoidine provides a potential alternative accessible through the use of environmentally benign routes in engineered bacteria and yeast^11–15^. High-throughput approaches for biosynthetic pathway bioengineering to improve productivity will be critical to generate final strains that meet industry production standards.

## Results and discussion

### Microfluidic chip design

The microfluidic chip was designed to perform multiple functions, including the loading of droplets from an external liquid-handling instrument such as an acoustic printer or droplet generator into an oil layer, the on-demand mixing of droplets by electrowetting, and the electroporation of cells within the droplets. Reagents are introduced into the chip by dispensing droplets, kept separate until ready to mix, and mixed on demand by merging droplets by electrowetting, and cells in the droplets are transformed by on-chip electroporation. Additional reservoirs allow recovery incubation and screening.

**Fig. 2:**
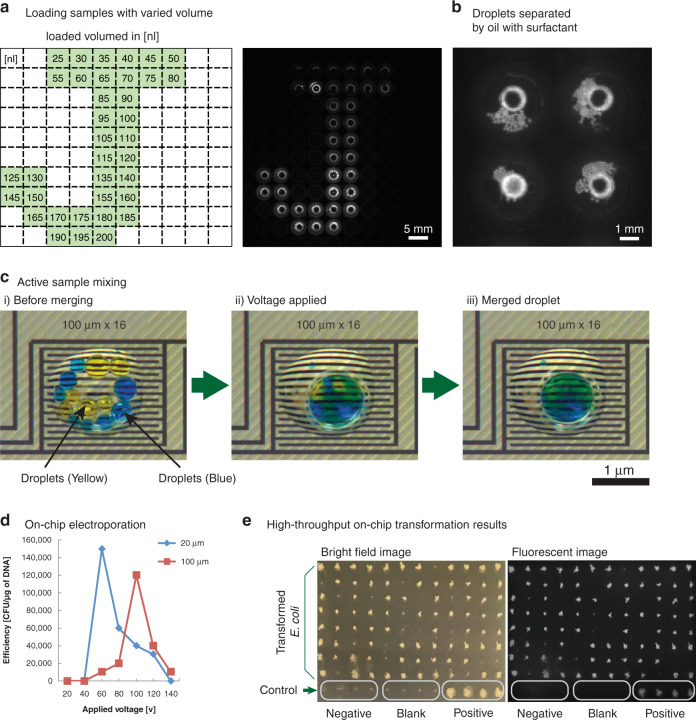
The microfluidic chip is capable of loading/mixing/electroporation in each chamber, enabling 100 discrete reactions on a single chip. **a** Sample volumes and loading sites can be programmed as desired. Green squares show where droplets were dispensed, and the numbers show the volume dispensed in nL. **b** Each chamber can be loaded with droplets containing different samples, and the droplets are suspended in oil containing a surfactant to prevent evaporation and accidental merging. **c** Electrowetting enables on-demand sample mixing by merging droplets. **d** The optimal voltage required for maximum transformation is dependent on the gap between electrodes. In the final chip design, we used 100 µm gap electrodes. **e** One hundred parallel electroporation reactions achieved over 80% successful transformation of *E. coli* with GFP plasmids. GFP was expressed by induction with IPTG and confirmed by measuring the fluorescence intensity (excitation/emission = 460 nm/515–535 nm)

The first step in a high-throughput operation is the introduction of a large number of samples in parallel. We designed the microfluidic chip layout to conform to 384-well microtiter plates for a scalable approach (chips can be designed and made in 1536-well format with minimal incremental cost) for loading reagents. This loading is facilitated by the integration of the chip with a commercially available robotic loader. We use a 3D printed holder (Fig. 1 and Supplemental Design Data) to align the location of the chip connecting ports to match the jetting locations of a Labcyte Echo 550 acoustic printer. This approach, however, is general and does not require a specific instrument: it can be used with other robotic liquid handlers, acoustic printers, droplet injectors, piezoelectric inkjet printers, or electrospray depositors. Integration with an automated loader allows the dispensing of small (200 nm) droplets directly into the microfluidics chip. The volume of reagents injected into each well is precisely controlled by the number of droplets injected into the wells, as shown in Fig. 2a. The addition of reagents *via* droplets enables on-demand mixing- the oil contains a surfactant to keep droplets separate, and the actuation of electrodes lowers the surface tension, permitting droplets to merge for reagent mixing (Fig. 2b, c and Supplementary Movie 1). On-demand mixing obviates the need for manual premixing of competent cells and DNA. The oil phase also prevents evaporation, a major issue in traditional low-volume microtiter plates. While the droplet can be merged with a simple pulse of direct current (DC) voltage with a duration of 10–100 msec, it is preferable to apply alternating current (AC) voltage to prevent electrolysis of the aqueous medium. Based on previous reports, we used an AC frequency of 80–100 kHz with a 10–100 msec duration for manipulating and merging droplets^6,7,10^.

### Electroporation in a microfluidic chip

Cell electroporation is potentially a more efficient process on a microfluidic chip than in cuvettes or microtiter wells because the micrometer size of the droplet is similar to the cell diameter. The micrometer size of the droplets also makes diffusion-based mixing faster. We tested different electroporation conditions by fabricating microfluidic devices with two different electrode gaps, 20 µm and 100 µm, and performed transformation with different electroporation voltages for a GFP (green fluorescent protein)-expressing plasmid in *E. coli*. One side effect of electroporation is electrolytically created bubbles at the anodes (Supplementary Fig. 1 and Supplementary Movies 2, 3). The size and number of bubbles increased with higher voltage. Shear stress from bubble formation can cause cell lysis^16^. There is an optimal point for electric field magnitude: too little and no transformation happens, too much and the cell undergoes lysis because of the high field and/or bubbles. Our platform allows us to find the optimal electroporation condition in one experiment by applying different electric fields in different wells, taking advantage of the feature that each well is individually addressable. Electrodes with 20 µm and 100 µm gaps reached maximum efficiency at 60 V and 100 V, respectively (Fig. 2d).

Our microfluidic chip configuration with individually addressable electrodes permits the multiplexing of electroporation (Supplementary Fig. 2), a feature not available in traditional cuvette-based electroporation (Table 1). Available multiplexed electroporation apparatuses (e.g., BTX High-throughput 96-well electroporator) are not practical for multiplexing because a single arcing event results in failure of the entire row of electroporation. Multiplexing enables large numbers of DNA edits at once under identical conditions or the same edits under different conditions, scaling the bioengineering process in a repeatable manner. Multiplexed electroporation, however, is difficult to perform on-chip for multiple reasons, including cross contamination and electrical wiring and footprint limitations. Previous efforts at multiplexing have relied on serial single-plex electroporation, such as flow-through devices with a single electroporation site^10,17,18^, or the number of parallel reactions has been limited to less than 10 due to physical constraints of the device configuration and electrical systems^8,19,20^. Our approach is inherently scalable and overcomes the cross-contamination issue by physically separating each reaction site in the microfluidic chip (Fig. 2a).

We experimentally verified our capability for multiplexed transformation by performing 100 electroporation reactions in parallel, including 4 negative transformations, and achieved over 80% successful transformation of *E. coli* with GFP plasmids (Fig. 2e and Supplementary Fig. 3). Since each electrode is individually addressable, each reaction chamber can be considered an individual transformation cuvette. This permits the testing of multiple combinations of strains/plasmids with the ability to produce up to 100 independent genetic modifications in one experiment.

### On-chip CRMAGE disruption of the *galK* gene

**Fig. 3:**
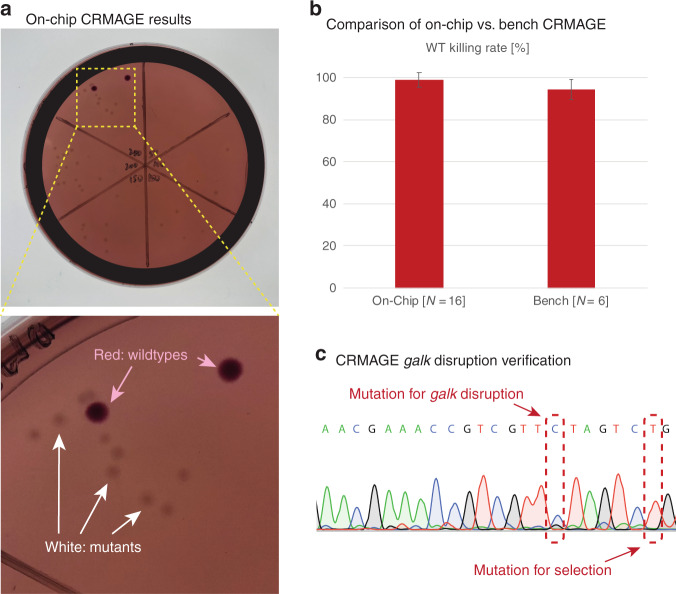
The microfluidic chip was able to use CRMAGE to disrupt *galK* with an over 98 ± 3% success rate. **a** Mutants from on-chip electroporation were plated on MacConkey agar plates: white colonies indicate successful *galK* disruption, while red colonies indicate the wild type. **b** The killing rate of the wild type (WT) is similar between the on-chip process and the benchtop process, with both exceeding the 90% success rate. Killing rates are calculated by counting the white colonies (i.e., mutants with *galK* disruption) as a proportion of the total number of colonies, including the red colonies (i.e., wild type). Error bars denote the standard deviation of biological replicates (*N* = 16 for the on-chip process and *N* = 6 for the benchtop process). **c** CRMAGE mutation for *galK* disruption was verified by Sanger sequencing. One of the mutations in the oligo produces *galK* disruption, while the other mutation is required for Cas9 selection since it is in the PAM region

We implemented CRMAGE^3^ (combination of CRISPR/Cas9 with ℷ Red recombineering) in our microfluidic platform. The Cas9 protein selects against cells that do not incorporate the supplied mutated DNA oligo into their genome through ℷ Red recombineering at the position indicated by the guide RNA (gRNA). The result is a population of cells displaying edited genomes at the points determined by the oligos and gRNA. To establish a proof of principle for on chip CRMAGE, we leveraged a classic genetic colorimetric screen. In this screen, a previously characterized point mutation to *E. coli*’s native galactokinase gene, *galK*, prevents the strain from growing on galactose medium^21–23^. When plated on MacConkey agar plates containing 1% galactose, WT strains capable of galactose fermentation turn red (no genetic change), while mutant strains are colorless (white, successful genetic change) (Fig. 3a). This colorimetric screen allowed us to rapidly screen the results of our engineering. CRMAGE targeting *galK* was optimized by fine tuning the concentration of the gRNA and the Cas9 inducer anhydrotetracycline (Supplementary Fig. 4), yielding a 98 ± 3% rate of disrupting *galK* using on-chip transformation vs. 94 ± 5% in a bench process (Fig. 3b). These results statistically represent the same success rate. We believe that the slightly higher mean of on-chip transformation compared with the benchtop process is due to the larger volume loss for benchtop cuvettes while transferring the cells for recovery. This represents an additional advantage of the on-chip transformation. As a confirmation of the colorimetric screen, *galK* mutants were also verified by Sanger sequencing (Fig. 3c). Our results show that CRMAGE can effectively be used on a chip to introduce point mutations, increasing the feasibility of a high-throughput CRMAGE platform.

### On-chip pathway optimization of an indigoidine-producing strain

**Fig. 4:**
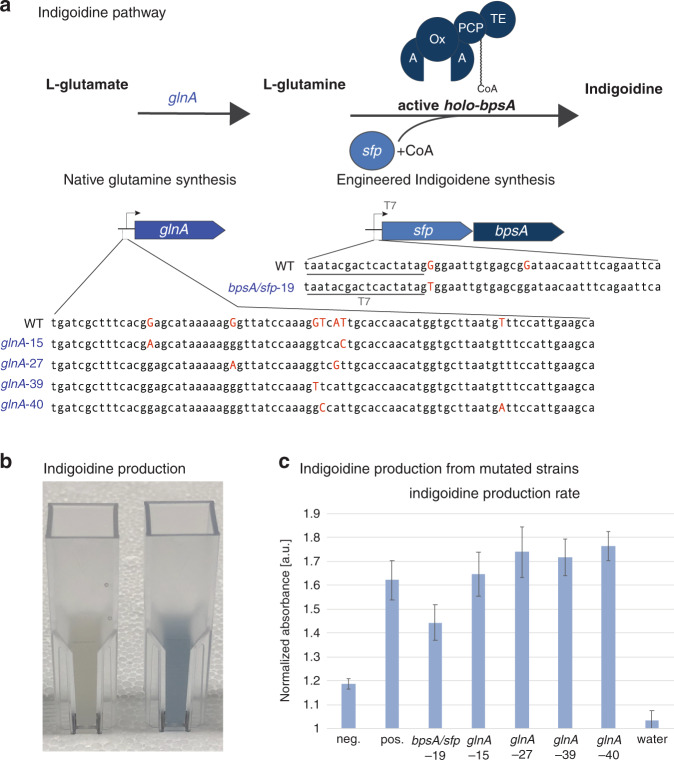
The microfluidic chip allows automated genome modification resulting in indigoidine production changes. **a** Two genetic targets in the indigoidine pathway (*bpsA/sfp*), and four targets that affect the supply of glutamine, a precursor for indigoidine production (*glnA*), were selected. **b** Indigoidine-producing strain in the right cuvette produces a blue color with the highest absorbance at 615 nm. **c** The genetic modifications impact the production of indigoidine (quantified by normalizing absorbance at 615 nm by 800 nm to minimize any background noise). Error bars denote the standard deviation of biological triplicates

To demonstrate the utility of microfluidic CRMAGE, we applied the method to modify an *E. coli* strain to produce indigoidine. To generate a stable, indigoidine-producing *E. coli* strain, we chromosomally integrated the heterologous pathway genes *bpsA*, encoding the nonribosomal peptide synthetase (NRPS) that converts glutamine to indigoidine, and *sfp*, a 4′-phosphopantetheinyl transferase required for the activation of *bpsA* under control of the strong, inducible T7 promoter (Fig. 4a)^11,12,24^.

We chose six genetic targets for point mutations (Supplementary Table 1): two targeting the engineered pathway (*bpsA* and *sfp*) and four targeting the native glutamine pathway (*glnA*), which is the precursor to indigoidine. These sites were selected because they have a PAM site downstream and relatively few off-target sites. The results of these genomic modifications were verified by Sanger sequencing (Supplementary Fig. 5) and were reflected in the final production of indigoidine (Fig. 4b). The modification in the native glutamine pathway in the *glnA*-40 mutant resulted in an increase in production, whereas the modifications in *glnA*-27 and *glnA*-39 resulted in a smaller (nonsignificant) increase. The *bpsA*/*sfp*-19 mutant caused a decrease in production, and the *glnA*-15 mutant resulted in no change (Fig. 4c).

## Conclusions

We developed a droplet-based microfluidic system capable of 100 transformations in parallel. Transformation conditions were optimized by the expression of GFP in *E. coli* using electrodes with different gaps as a function of applied voltage. We adapted the CRMAGE gene-editing protocol for point mutations, and on-chip mutations disrupting *galK* achieved a >90% success rate, comparable to the results obtained with the benchtop protocol. As a demonstration of our platform to optimize biosynthetic pathways, we produced 6 different mutations of indigoidine-producing *E. coli* using CRMAGE.

While we demonstrated 100 parallel reactions and 6 different mutations in this chip, the number of reactions is limited only by the area of the current chip. Typical semiconductor manufacturing processes use 8 inch or 12 inch wafers, and our current chip requires an effective footprint of 2 × 2 inches for 100 reactions, enabling the fabrication of up to 1600 reaction sites per wafer. Independently addressable reaction wells also enable the optimization of electroporation conditions for different strain/plasmid combinations. Our chip is also compatible with species other than *E. coli* (e.g., yeast), making it a generic bioengineering platform.

The automated platform for multiplexed transformation holds the promise of accelerating the design-build-test-learn cycle and optimizing biosynthetic pathways. This technology could provide the technological basis for self-driving bioengineering labs^25–27^, which couples automated experimentation and AI systems that propose experiments and gauge the resulting data to accelerate the bioengineering process^28,29^.

## Materials and methods

### Materials and fabrication

For the fabrication of the devices, we purchased transparent film photomasks printed at Fine Line Imaging (Colorado Springs, CO), reagents including SU-8 5, SU-8 2075, S-1811 and SU-8 Developer from Microchem (Newton, MA), gold-coated glass substrates and chromium-coated glass substrates from Telic (Valencia, CA), indium tin oxide (ITO)-coated glass slides from Delta Technologies (Stillwater, MN), MF-321 positive photoresist developer from Rohm and Haas (Marlborough, MA), CR-7 chromium etchant from OM Group (Cleveland, OH), AZ-300T photoresist stripper from AZ Electronic Materials (Somerville, NJ), Aquapel from TCP Global (San Diego, CA), and poly (dimethylsiloxane) (PDMS) from Dow Corning (Midland, MI).

Microfluidic chips were fabricated using lithography processes. First, we patterned electrodes for electroporation and electrowetting on metal-coated glass substrates using a mask aligner (OAI, Model 200). Photoresists (S-1818, Shipley) were developed with MF-321 developer after 5 s of UV exposure, and then electrodes were etched with acids: gold etchant, CR-7 chromium etchant, or hydrochloric (HCl) acid for ITO. The electrodes had a thickness of ~100 nm after etching, and residual photoresist was stripped with an AZ stripper. We hard-baked the substrates at 150 °C for 15 min and then cleaned the surface with oxygen plasma (SERIES 800 MICRO RIE, Technics) for 2 min. Next, SU-8 3005 was spin coated with a spinner (CEE Model 100 Spin Coating System, Brewer Science) to obtain a 5 µm thickness of the dielectric layer. The windows for electroporation were patterned with a mask aligner and developed with SU-8 developer.

For the chamber layer, we used medical grade pressure-sensitive adhesive (PSA) with double-sided adhesives (Medical Tape, 3 M) ~100 µm in thickness. The PSA layers were cut into chambers using a stencil cutter (Cameo 3, Silhouette). For the top layer, we utilized ITO-coated PET film (Sigma–Aldrich). Connecting ports with a diameter of 1 mm were milled with a computer numerical control (CNC) milling machine (Othermill, Bantam Tools). After aligning the PSA layer with the bottom substrates under a microscope, the top PET film was placed onto the PSA layer. For the incubation chamber, the PDMS slab was punched with a 3 mm diameter puncher (Harris Uni-Core, Ted Pella).

For the biological samples and reagents, we purchased electrocompetent *Escherichia coli* (*E. coli*) cells (MG1616) from New England Biolabs (Ipswich, MA). We obtained plasmids (GFP plasmids, pZS4Int-tetR, pMA7CR_2.0, and pMAZ-SK) for *E. coli* from a public registry at the Joint BioEnergy Institute (https://public-registry.jbei.org/folders/610)^30^.

### Microfluidic chip design and operation

Figure 1 shows the design of the chips with 100 reaction sites. Microfluidic chips consist of four layers: (1) glass substrates with patterned electrodes for electroporation and DMF manipulation, (2) a PSA layer for the reaction chambers, (3) a top layer with ITO electrodes and connecting ports, and (4) PDMS chambers for additional incubation medium (Supplementary Fig. 6). The reaction chambers are prefilled with oil, and droplets containing biological samples are loaded onto the chambers with external sample sources. Applying voltage to the electrowetting electrodes initiates the mixing of droplets, and electroporation is performed by applying an exponential decay pulse to the pairs of electrodes in contact with the droplets. After electroporation, droplets can be incubated on chip by immediately introducing the recovery medium or can be introduced to the larger volume of recovery medium in 96 plates^31^.

Liquid samples are loaded onto microfluidic chips in the form of droplets containing biological parts. Droplets with the same size scale as the microfluidic channels (nanoliter~microliter) are dispensed onto the substrates (e.g., glass, PDMS, printed circuit board (PCB), etc.) of microfluidic devices either directly or remotely from the sample source (e.g., robotic liquid handler, acoustic printer, droplet injector, piezoelectric inkjet printer, electrospray deposition). Dispensing could be achieved by targeting the microfluidic devices either directly in the air environment or through connecting ports in the oil environment^32^. The sample loading process was successfully demonstrated to load multiple aqueous samples onto a presealed device via the connecting ports without merging (Supplementary Fig. 7). Supplementary Fig. 8 shows the fabricated microfluidic chip with a 3D printed holder to align the location of the connecting ports to match the jetting locations.

### CRMAGE gene-editing

#### CRMAGE protocol

We utilized a modified version of the CRMAGE protocol developed by Ronda et al.^3^, and its simplified process flow is shown in the subset of Fig. 1. Then, 100–200 ng of pMAZ-SK plasmid and 0.5 µL (10 pmol/µL) of oligo were added to 50 µL of electrocompetent cells on ice. Electroporation pulses were applied using a commercial electroporator (Bio–Rad, Gene Pulser Xcell, and BTX, ECM-350) with the following settings: voltage = 1800 V (for benchtop), resistance = 200 Ω, capacitance = 25 µF. Immediately after electroporation, the transformed cells were transferred to 1 mL of Lennox recovery medium with 100 μg/mL carbenicillin (to maintain pMA7CR_2.0) and 35 μg/mL chloramphenicol (to maintain pZS4Int-tetR) and incubated for recovery at 37 °C with shaking. After 2 h of recovery incubation, kanamycin was added to reach a concentration of 50 μg/mL, and the culture was incubated for an additional 3 h for selection. After 3 h of selection incubation, anhydrotetracycline (atet) (200 ng/mL) was added, and the cells were grown overnight at 37 °C with shaking. Then, the cells were plated on agar plates with 50 μg/mL kanamycin, 100 μg/mL carbenicillin, and 35 μg/mL chloramphenicol and incubated at 37 °C until the colonies became visible, which typically took ~12 h.

#### CRMAGE targeting loci for indigoidine pathway optimization

We adapted the on-chip CRMAGE process to target indigoidine pathways in *E. coli*. We engineered a wild type strain (MG1655) to chromosomally express the genes required for indigoidine production (*bpsA* and the activator protein *sfp*) from the T7 promoter, a very strong and commonly used promoter in synthetic biology. We designed oligos and gRNA sequences for CRMAGE plasmids targeting these T7 promoters, aiming to modify the gene expression of both pathway genes (*bpsA* and *sfp*) simultaneously. Additionally, we sought to investigate the impact of changing the availability of the indigoidine precursor glutamine on the production of indigoidine. Thus, we included mutations targeting the native promoter that drives glutamine synthetase (*glnA*), which converts glutamate into glutamine. The designed set of oligos and gRNA sequences is shown in SI Tab. 1. The first two sets target *bpsA/sfp*, and the other four sets target *glnA*. Following the CRMAGE guidelines from Ronda et al., the indigoidine-producing strain, modified MG1655, was mutated. These mutations were screened by antibiotics (kanamycin), and selection was subsequently performed with atet-induced Cas9 and gRNA expression. Indigoidine was produced by induction with isopropyl β-D-1-thiogalactopyranoside (IPTG). Induced cells were incubated at 30 °C at 200 rpm for optimal production conditions. Since indigoidine has a strong peak absorbance at 615 nm, the production rate can be quantified by measuring 615 nm absorbance with a spectrophotometer, as shown in Supplementary Fig. 9. The results clearly indicate the successful production of blue pigment. The indigoidine production rate for each mutant was quantified by normalizing the 615 nm absorbance to the 800 nm absorbance to minimize any background noise from unrelated molecules and to prevent pipetting errors in volume.

#### CRMAGE target selection

To create CRISPR gRNA, the entire JBEI-19353 strain genome was searched for PAM (NGG subsequences) on both the forward and reverse strands, which are within the neighborhood of the desired genome modification site. The neighborhood in this case was chosen to be an interval of 80 bases (the size of the repair template). Upon the identification of a PAM site, a corresponding N20 was constructed as a 20-base region of homology with the complementary strand. These N20 sequences were then scored by performing a homology search against the rest of the genome using a fuzzy regex search that allowed an off-target match when the N20 deviated by fewer than 2 bases from the recognized target sequence. Six sequences were selected with the fewest off-target matches found in the rest of the genome. After the design of the N20 sequences, repair templates 80 bases in length with the desired change integrated into them were created. The full code can be found in the supplementary Jupyter notebook “MakeCRISPRgRNA.ipynb”.

## Supplementary information

Supplementary Information

Supplementary Design Data

Supplementary Movie 1

Supplementary Movie 2

Supplementary Movie 3

MakeCRISPRgRNA.ipynb
